# Non-Destructive Health Monitoring of Structural Polymer Composites: Trends and Perspectives in the Digital Era

**DOI:** 10.3390/ma15217838

**Published:** 2022-11-06

**Authors:** Salim Chaki, Patricia Krawczak

**Affiliations:** IMT Nord Europe, Institut Mines-Télécom, Univ. Lille, Centre for Materials and Processes, 941 rue Charles Bourseul, 59508 Douai, France

**Keywords:** polymer composites, non-destructive testing (NDT), structural health monitoring (SHM), damage detection, durability

## Abstract

Non-destructive testing (NDT) techniques are usually used for the characterisation of defects arising in polymer composites during manufacturing or in-service use. However, each of these NDT techniques cannot always allow a full diagnosis of the material’s or component’s structural health. Thus, several techniques have to be combined in order to improve the diagnosis of the damaged state of composite structures and their evolution during the part’s life span. This opinion paper proposes a critical overview of the use and applicability of these NDT techniques for the detection and characterisation of damage to structural composite materials in view of in-service performance assessment and residual durability prognosis. It also addresses some current trends of structural health monitoring (SHM) of these materials, such as sensor–actuator embedding and NDT data fusion, and draws future perspectives on how composite SHM could evolve in the digital era, taking advantage of artificial intelligence, Internet of Things and big data to implement digital twins.

## 1. Introduction

Structural composite materials (i.e., fibre-reinforced polymer composites) are widely used in various industrial fields (railway vehicles and infrastructure, wind turbine blades, pressure vessels, etc.) because these materials are lightweight, resistant to corrosive environments and exhibit remarkable mechanical and physical properties. However, more than five decades after their industrialisation, their widespread acceptance as a reliable class of engineering materials remains an issue. Indeed, the manufacturing of these materials is critical and almost always induces the appearance of some defects such as fibre misalignments, porosities, etc. Under mechanical loading, internal and multi-scale damages may be initiated from these manufacturing defects, such as matrix cracking, fibre pull-out or interfacial debonding between the fibres and the matrix, fibre breakage, and delamination between plies in laminated composites, which are all invisible from the material outside. Additionally, some potentially dangerous surface defects may appear such as projectile impacts, scratches and emerging micro-cracks directly accessible but not always visible to the naked eye.

Non-destructive testing (NDT) methods have proved to be invaluable both in detecting the initial defect and monitoring the damage process of composite materials under mechanical loading (i.e., in situ) or after mechanical loading (i.e., ex situ). The most commonly used NDT techniques are visual testing (VT), dye penetrant testing (PT), ultrasonic testing (UT), X-ray radiography (RT) and tomography (µCT), infrared thermography (TT), and acoustic emission (AE). Depending on the damage mechanism involved, the part geometry and the in situ conditions of use, one NDT technique will be preferred over another, or several techniques will be combined to improve the damage diagnosis of composite structures [1,2,3]. Indeed, Structural Health Monitoring (SHM) of composite materials involves several economic and security challenges. For instance, the results of non-destructive testing allow for making major decisions regarding the conformity of produced parts (scrap rate), requalification of risk-prone structures and authorisation for operation (or not) in industrial machinery and systems. The consequences of a wrong diagnosis can be purely economic if the rejected part is healthy but far more serious if a defective major component of risk-prone equipment is kept in service. Thus, it is crucial to implement a suitable monitoring process for each testing configuration to obtain sufficiently precise and discriminating results.

This paper proposes a critical overview of the use and applicability of the most popular NDT techniques for the detection and characterisation of defects and mechanical damages occurring in composite materials. A more complete characterisation of the damage is suggested by using several different NDT techniques in situ and either in real-time or periodically. Some guidelines and future perspectives are proposed on how SHM of composite materials could evolve in the digital era, taking advantage of artificial intelligence, data fusion and big data.

## 2. Physical Principle of Non-Destructive Testing

In all NDT methods, the detection of a defect in a part amounts to highlighting a material discontinuity within it, which can be illustrated by a local variation of one or more physical or geometric properties that are detrimental to its proper operation. Numerous NDT methods exist, based on different physical phenomena, including acoustic waves (ultrasound, acoustic emission, etc.) or electromagnetic waves (infrared thermography, X-ray radiography, etc.).

**Figure 1:**
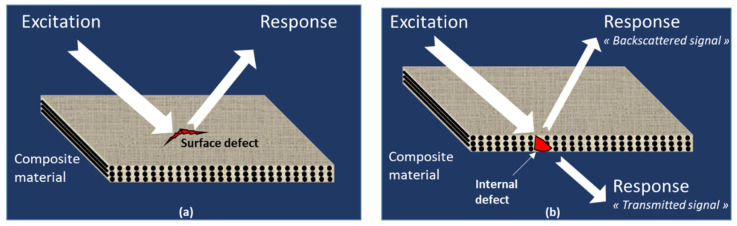
Non-destructive techniques principles: (**a**) surface techniques, (**b**) volume techniques.

Generally, all NDT methods have a similar operating principle, which is based on the excitation of the tested material by an energy flux and the reception of its response, for analysis, as reflected or backscattered or transmitted signals after the interaction, respectively, with its geometry or its microstructure including the internal defects. Two categories of NDT can be distinguished, namely surface and volume diagnoses (Figure 1). The use of surface or volume analysis depends on the type of defects, namely surface or internal defects. For each category of defect, different NDT techniques can be used depending on the material, its geometry and the in situ conditions of use. Particularly, composite materials generally allow poorer flaw resolution than homogeneous materials because of the greater contribution of noise from matrix-additive (fibre or particle) boundaries. Thus, it is necessary to implement a suitable NDT technique for each application to obtain sufficiently precise and discriminating results.

## 3. Surface Non-Destructive Testing Techniques

Roughness, scratches or blistering located on the surface of a composite material can be induced by impacts or chemical aggressions. Therefore, it is necessary to detect and characterise these types of defects using surface NDT techniques.

### 3.1. Visual Testing

The common-sense approach to NDT is to first inspect visually for surface flaws (mark-off, bow waves, wrinkles, etc.) that can often be seen by careful direct visual inspection [4,5,6]. Normal eyesight is often sufficient to indicate where impact damage or delamination beneath the surface has occurred. For translucent composite materials, such as glass fibre reinforcing matrix epoxy, it is possible to detect porosity and the fibre matrix bond condition down to a depth of 10 mm. However, optical aids such as magnifying glasses, illumination techniques, miniature video cameras and small diameter endoscopes, etc. should be used wherever possible to enhance inspection details and to provide digital images for post-processing, traceability and archiving.

Thanks to digital imaging, some studies recently proposed to exploit deep learning for quantitative assessment of visual detectability of different types of in-service damage in laminated composite structures such as aircraft and wind turbine blades [7]. Interestingly, photogrammetric point cloud analysis using remotely sensed point cloud data also possible to track full-scale 3D infrastructure deformation at the millimetre scale in a field environment [8].

Visual testing is an inexpensive, simple and rapid method of detecting surface defects. However, it will miss any sub-surface flaws that do not cause a surface disturbance and may miss barely visible impact damage. Therefore, it cannot be used on its own as an inspection technique; it must be supplemented by other deeply penetrating methods.

### 3.2. Dye Penetrant Testing

**Figure 2:**
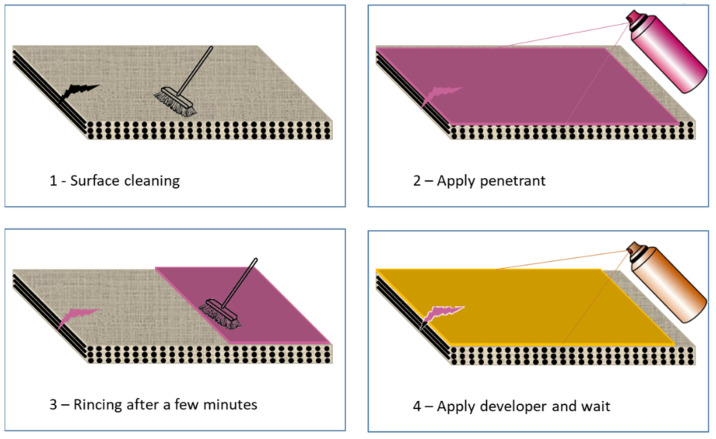
Principle of dye penetrant testing: (1) surface cleaning, (2) apply penetrant, (3) rincing after a few minutes, (4) apply developer and wait.

Dye penetrant testing is a widely applied and low-cost inspection method used to check surface-breaking defects in all non-porous materials. A dye is sprayed on the composite and it gets drawn into the cracks and pores because of surface tension. After a certain dwell time, the composite is wiped clean and a developer is applied. The developer, usually a dry white powder, draws out the penetrant from the cracks and pores so that the visual inspection can then be performed (Figure 2).

This inspection method is widely used in the NDT field in almost all industrial sectors, both in manufacturing and maintenance. For instance, penetrant inspection dye was used on a composite sample under tensile loading [9] in order to improve the observation of interlaminar damage initiation. It was also carried out to investigate the extent of surface/interlaminar damage in the top layers due to the low-velocity impacts of different energies [10,11]. Machine learning methods, such as Random Forest (RF), were used to perform automated defect detection for fluorescent penetrant inspection [12,13]. Results of this work, obtained on a titanium material but applicable to composite materials, indicate that RF is able to correctly identify 76% of defects with a false call rate of 0.42, demonstrating a capability comparable to that of a human operator [12].

Dye penetration inspection is defined as a global method allowing for the control of a part in a one-shot operation, or for treating a large series of small parts at the same time, at lower costs and with very good reliability. However, it does not allow for the evaluation of the depth of the defects and has a significant impact on the environment due to the use of toxic and flammable products.

### 3.3. Thermography

**Figure 3:**
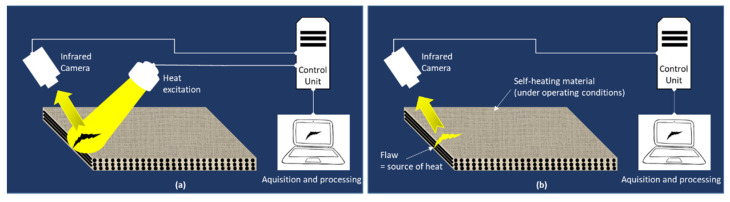
Principle of active (**a**) and passive (**b**) infrared thermography.

Infrared thermography is an interesting alternative inspection method to dye penetrant testing that is more respectful of the environment. Defect detection consists of collecting the radiation included in the spectral band [2–15 µm] via an infrared camera, naturally emitted by a composite part due to its in-service operation (passive thermography) or artificially by stimulating its surface by an external heat source (active thermography) (Figure 3). Depending on the material nature, its thickness and the defect depth position, different types of excitation using transient or continuous heat sources can be used to thermally stimulate the part to be inspected. The presence of defects disrupts the normal pattern of heat flow that would be expected in a sound part.

In the literature, passive thermography was widely used to evaluate the in situ damage that occurred in composite materials under continuous mechanical loadings [14,15,16] or transient impacts [17]. The latter author operated in the frequency domain for a better understanding of the dynamics of crack formation as well as accurate quantification of damage extent in situ during impact testing of thin carbon–epoxy composite materials [17]. Under static tensile loading of a glass–epoxy composite, this technique was used to evaluate the created damage, where fibre/matrix debonding caused short and intense heat dissipation, whereas delamination induced more extended spatial and temporal heat dissipations [14]. It was also used to monitor heating from damage formation in a hat-stiffened woven graphite epoxy composite panel during quasi-static seven-point load testing [15]. Self-heating behaviour analysis under fatigue loading of composite materials is a new way to determine the material’s fatigue limit [18,19,20,21]. The major advantage of the latter measurement is the reduction of the time and cost of the experimental study.

Active thermography requires thermal excitation by using, for example, pulsed [22] or continuous lamps [23] or even acoustic vibration of the tested structure (vibrothermography) using a high-power ultrasonic source (sonotrode) [24]. A correlation between the material cooling speed after heating by lamps and the applied tensile stress levels was obtained for a glass–epoxy composite [23]. Regarding the obtained results, this damage indicator is not sufficiently sensitive to the damage (barely 30% at failure). Porosity detection can be performed using lock-in thermography, which consists of acquiring infrared pictures while the specimen surface is thermally stimulated with a sinusoidal heat flux [25]. However, in-depth characterisation is still limited, depending on the sinus frequency, where only subsurface (no deeper than 3.5 mm) millimetre size defects could be visible [26,27]. Recent advancements in the field of artificial intelligence can efficiently support the post-processing of thermographic data without any human (inspector) intervention [28,29].

Generally, thermography testing is more sensitive to flaws near the surface. However, cracks that are aligned parallel to the direction of heat flow may go undetected. Moreover, the main limitation in applying thermography to composite inspection is the anisotropy that produces different thermal properties in different directions. The main advantages are the eco-friendly and contactless aspects of the method that can be used several metres from the specimen allowing remote sensing.

### 3.4. Shearography

**Figure 4:**
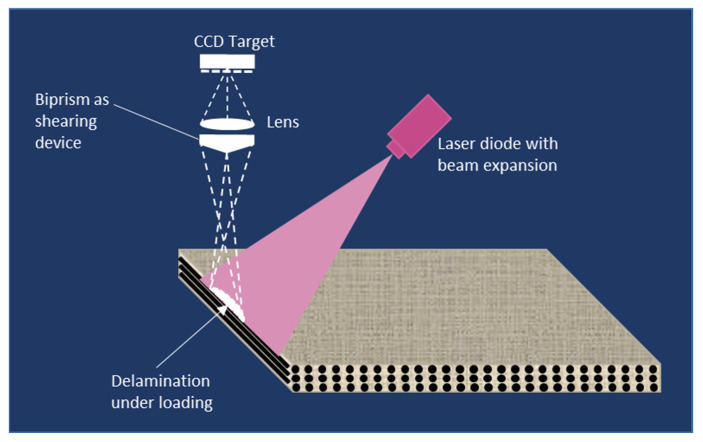
Principle of shearography.

Under loading, a near-surface defect will decrease a composite part’s local strength and therefore its surface will deform differently. These differences are very small, therefore, a technique based on optical interference, such as shearography (Figure 4), is suitable to inspect structures by looking for defect-induced anomalies in the surface strain field, from the fringe pattern and/or phase map [30].

Shearography provides wide-area qualitative imagery of in- and out-of-plane displacement variations on the surface of a structure under loading. The used loading for defect detection includes thermal, pressure, vibration, microwaves, and so on [31]. Acoustic waves (50 kHz) were used through piezoelectric transducer excitation of the sample (carbon fibre composite) to defect imaging by the named acoustic shearography technique [32]. Moreover, shearography is widely adopted to evaluate aeronautic composite parts, especially to detect debonding or the beginning of delamination [33,34,35]. Its potential for the detection and localisation of different types of defects (calibrated and real defects) in 4 mm-thick [0, ±45, 90]_s_ laminates was demonstrated, with good agreement with the real description of the studied defects [35]. For thick composite materials (more than 50 mm thickness), shearography also demonstrated the capability of detection and localisation of defects such as flat bottom holes with thermal loading stimulation [36,37]. A simulation dataset and hybrid training, based on digital shearography images, in deep learning, were performed for defect detection in various kinds of materials (epoxy carbon, E glass fibre, ABS thermoplastic, etc.) [38]. The results showed that a simulation dataset, generated without any real defective specimen, shearography system or manual experiment, can greatly improve the generalisation of a deep learning network when the number of experimental training images is small.

Shearography allows for non-contact inspections to be conducted rapidly and with a high degree of sensitivity, especially for field measurements where the conditions are far from ideal. Because the specimen must be loaded to reveal any defects, there is always a possibility that damage might be incurred as a result of the testing procedure; however, in practice, the required loads to produce fringe patterns are several orders of magnitude less than the working load of the structure. However, as shearography is an optical surface-based technique, the influence of lighting and the location of defects have a bearing on the resultant image. Moreover, the interpretation of shearography images is complex and requires extensive experience.

### 3.5. Digital Image Correlation

**Figure 5:**
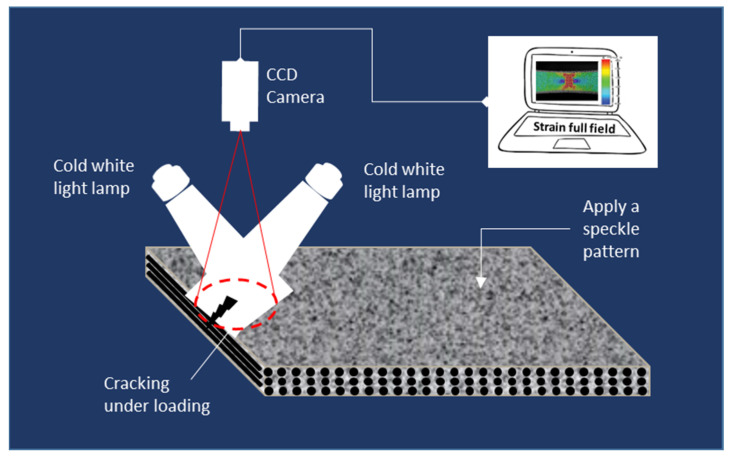
Principle of digital image correlation.

Digital image correlation (DIC) (Figure 5) is a non-contact optical technique that was used to measure in-plane (2D) or out-of-plane (3D in stereo-correlation) full-field strains on the surface of a sample under static or dynamic mechanical loads [39,40,41]. It does not directly provide insight into microscale and in-depth damage mechanisms; however, it can efficiently indicate zones of high-strain localisation that would correspond to mesoscopic or macroscopic damage locations in composite materials.

Digital image correlation was used to investigate the quality of adhesive joints between multi-layer composite panels where the crack initiation in the joint was correlated to the highest strain levels generated by applied shear loading [42]. It was also used to monitor mode I propagation in adhesively bonded joints while determining the crack-tip position and the extension of the cohesive process zone [43]. Furthermore, the method was widely used during fatigue tests to measure the evolution of the kinematic deformation energy per cycle of composite materials [21,44,45]. Moreover, full-field strain analysis was also useful in assessing the mechanical failure mechanism of core-shell hybrid composite rods [46]. A convolutional neural network (CNN)-based image semantic segmentation technique is proposed for pixel-level classification of DIC strain field images obtained for CFRP-laminated composites [47].

The biggest advantage of the method, over traditional strain measurement techniques such as strain gauges, is that it is a non-contact method allowing full-field measurement. However, one of the main drawbacks of the method is that it needs reference images at free stress conditions and it may be less accurate than traditional testing, especially if the camera is hit or moved during testing.

As a conclusion of this section, Table 1 summarises the surface non-destructive techniques’ potentialities in terms of sought flaws in composite materials and provides their advantages and limitations.

## 4. Volume Non-Destructive Testing Techniques

Internal defects in a composite material are generally not only invisible from the outside but also difficult to assess by conventional NDT methods. Depending on the material’s constitutive components, its architecture and the applied loading, the defects occur at different scales (nano-, micro-, meso-, macroscopic), their number can be very high and their distribution is multidirectional. These types of defects have different criticalities depending on their sizes, numbers, in-depth positions and service conditions. They can affect the proper operation of the composite part and reduce its lifetime. The most popular volume NDT methods are presented hereafter.

### 4.1. Ultrasonic Waves

**Figure 6:**
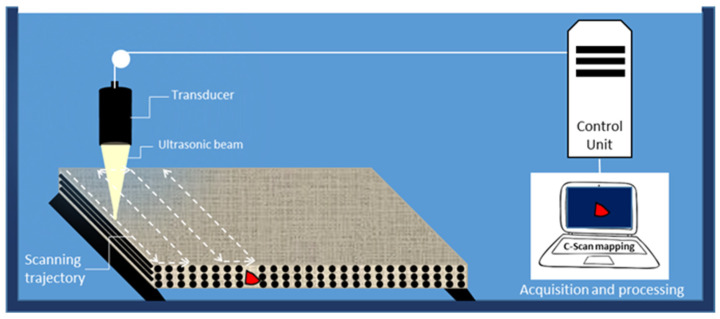
Principle of ultrasonic testing (C-scan example).

Ultrasonic testing is the most widely used non-destructive inspection method for the examination of composite materials (Figure 6). Most industrial applications use frequencies ranging between 0.5 and 5 MHz, depending on the material attenuation and the desired penetration depth. Three types of ultrasonic waves can propagate in media; volume waves, separated in compression (longitudinal) and shear (transversal) waves; surface or Rayleigh waves; and guided waves such as Lamb waves in plates [48]. Different types of transducers exist according to the testing configuration (contact, immersion, straight, oblique, plane, focused, mono-element, phased array, etc.). The operating modes include pulse-echo, through-transmission, back-scattering, time-of-flight diffraction (TOFD) and ultrasonic spectroscopy, which can be performed in contact or in immersion, or even in air coupling conditions.

The pulse-echo C-scan immersion technique was applied in the thickness direction of a cross-ply [0°/90°]_S_ glass—epoxy composite after different tensile stress load levels to monitor the damage induced perpendicularly to the loading direction based on density variation maps and image segmentation processing [49]. Most attempted inspections of thick materials were performed at lower frequencies (0.5–1 MHz) [50,51,52,53] and good results were obtained for CFRP composite laminates with a thickness of up to 15 mm [54]. However, thin composite materials were widely inspected using Lamb waves for damage detection [55] or material properties evaluation, such as the dynamic Young’s modulus [56,57]. Currently, phased array transducers are widely used for composite inspection thanks to higher speed scanning, higher resolution sensing and digital imaging obtained with this technology when compared to traditional transducers [58,59]. Nowadays, intelligent damage recognition of composite materials, based on artificial intelligence, machine learning, and deep learning, in ultrasonic testing results is largely used [60,61]. It allows for the avoidance of operator interpretation errors and reduces the time taken when analysing the results.

The ultrasonic method allows for the detection and characterisation of meso- and macroscopic defects (large porosities, impacts, delamination...) and cannot quantitatively assess the microscopic damage, which can only be characterised by an overall analysis of velocity or attenuation variations. The main technical issues associated with this method are attenuation, scattering and absorption of the waves, and also the shadowing effect in the case of multiple defects.

### 4.2. Acoustic Emission

**Figure 7:**
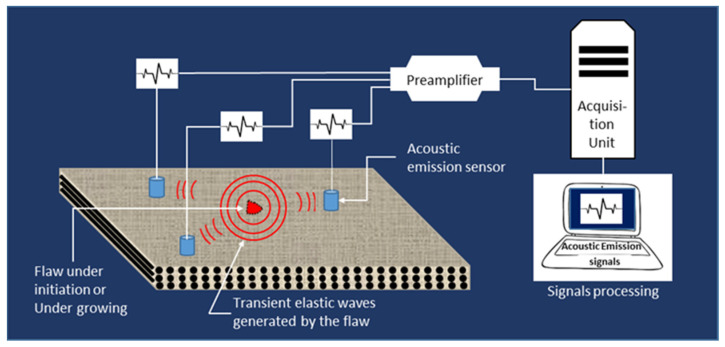
Principle of acoustic emission testing.

Acoustic emission testing is fundamentally different from other techniques using elastic waves as it relies on signals originating from the inside rather than from the outside of the specimen. These signals are generated from evolving defects resulting from mechanical, thermal or chemical solicitations of the material (Figure 7).

Acoustic emission is historically seen as one of the most efficient techniques to evaluate mechanical damage in composite materials. Damage monitoring can be performed in situ and in real-time, while analysing different parameters of the received acoustic emission signals, such as amplitude, cumulated counts, cumulated energy, duration, etc. [58,59,60,61,62,63,64,65]. This method is widely used in several industrial sectors, namely in the field of renewable energy systems and in particular for wind turbine blades [66]. Currently, the research works deal with the multivariable analysis of the acoustic emission parameters, such as principal component analysis, artificial neural networks, K-means, etc. in order to identify and classify damage mechanisms occurring in the material under loading [67,68,69]. For instance, the latter author used three multivariable analysis techniques (Principal Component Analysis (PCA), K-means and Kohonen Self-Organising Map (KSOM)) to characterise tensile loading damage of different glass fibre unidirectional composite samples ([0]_4_, [90]_4_), laminates [0/90]_S_ and neat epoxy resin [69].

Acoustic emission has many advantages, including the detection and localisation of evolving defects in situ and in real-time, and the prevention of industrial risks (predictive maintenance). However, the restriction to only evolving defects and the global and qualitative aspects of the monitoring are the main limitations of this method.

### 4.3. X-ray Radiography and Tomography

Radiography consists of the deployment of short-wavelength electromagnetic radiation (X-ray, γ-ray) to penetrate various materials, including composites, and forming a 2D image (radiograph) of the internal structure of the penetrated material. The basic principle is that parts of the specimen with different radiation absorption properties, such as defects, can be discriminated in the radiograph formed by the beam transmitted through the specimen. The method can be used to detect delamination, cracks and foreign inclusions, and also density and thickness variations [70,71,72]). Moreover, several studies indicate that the use of backscattered X-ray imaging is highly advantageous concerning the detection of water and moisture in carbon composite structures compared to other through-transmission techniques [73].

**Figure 8:**
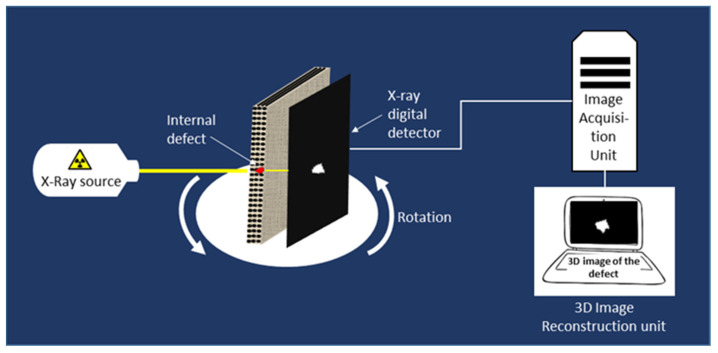
Principle of X-ray tomography.

X-ray tomography relies on the computerised reconstruction of a series of X-radiographs, which are collected by rotating the sample at a controlled angular step (Figure 8). The output data correspond to 3D images of characteristic elementary elements (known as voxels), which are coded in grey levels. Applying appropriate image post-processing protocols, such as grey level thresholding, banalisation, skeletonisation, geometrical filters, fibre segmentation algorithms, etc., makes it possible to discriminate between the matrix, the fibres, and the damage-induced voids or cracks [74,75,76,77]. Information regarding the operation, the advantages, and the drawbacks of different X-ray tomography systems including Synchrotron is provided elsewhere in the literature [76,77,78].

Some studies report that X-ray tomography is well adapted to investigate damage in glass–epoxy composites, whereas some restrictions exist for carbon–epoxy composites because the atomic numbers of the fibres and the matrix are relatively close. Some enhancements would be possible using a dye penetrant to improve the local absorbance contrast if the damage cracks are connected to the surface [79]. Currently, some modern data analysis, such as deep learning or machine learning, has been proposed to identify defects in composite materials in X-ray images [80,81,82].

The major drawbacks of these methods are their high cost and strict health regulations. Moreover, at the scale of large structures (typically of a few centimetres in length), only a cut portion can be analysed by X-ray tomography; consequently, this method becomes destructive.

As a conclusion to this section, Table 2 provides a summary of the potentialities of the volume non-destructive techniques in terms of sought flaws in composite materials as well as their advantages and limitations.

## 5. Hybrid NDT Analysis for Residual Lifetime Prognosis

A difficulty commonly encountered in NDT is the lack of a reference method that would be able to unambiguously evaluate total damage occurring in a composite material both on its surface and internally, as well as during or out of loading. Moreover, different damage mechanisms at different scales (nano-, micro-, meso- and macroscopic) can occur with an anisotropic distribution according to the material architecture and the applied loading. Thus, none of the NDT methods are solely capable of inspecting all types of defects occurring in composite materials, especially in in situ conditions and for complex parts. This is the reason why more and more authors [2,83,84,85] have chosen to implement multiple NDT techniques simultaneously to gather complementary information in order to improve the diagnosis of the damage state of materials.

**Figure 9:**
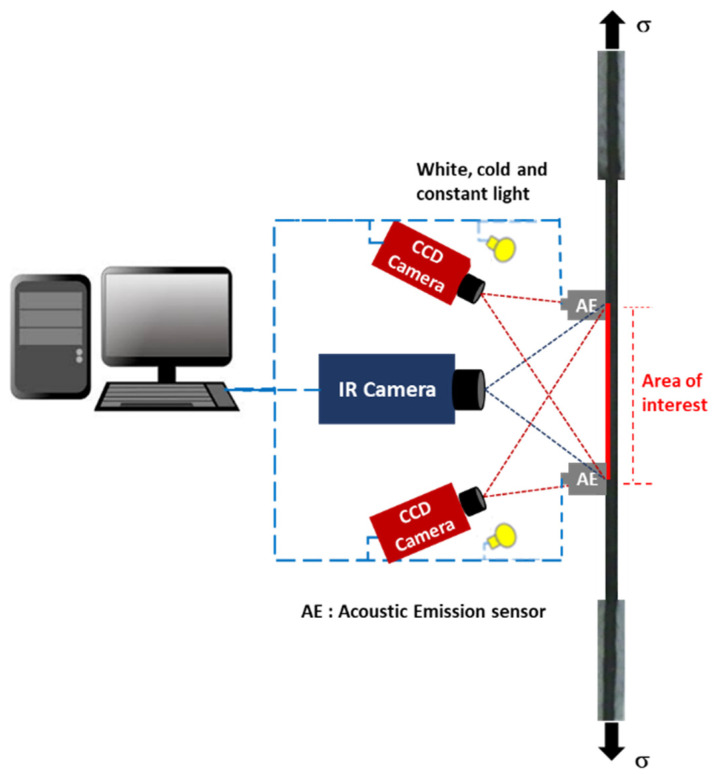
Example of a hybrid non-destructive testing system. Implementation of acoustic emission/ultrasonic guided waves, digital image correlation, and passive infrared thermography.

**Figure 10:**
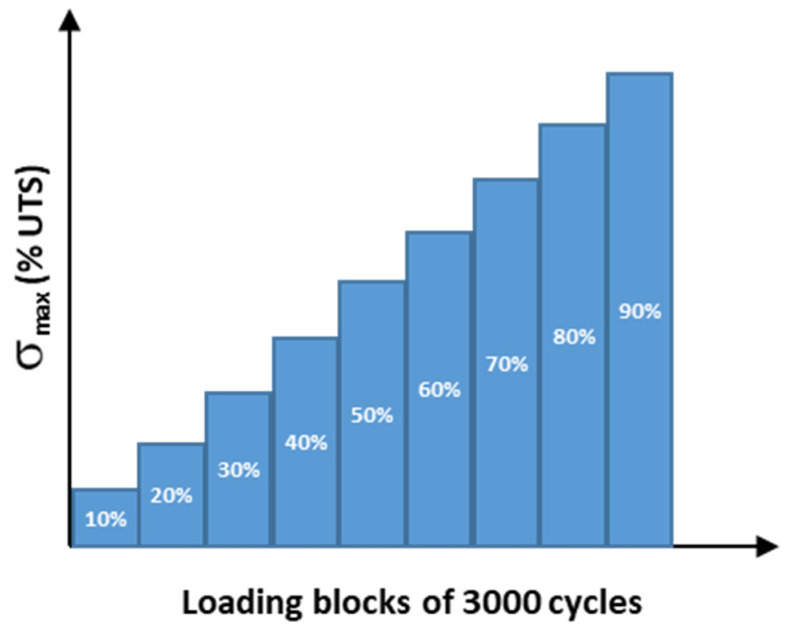
Applied fatigue loading profile during hybrid non-destructive testing procedure.

Figure 9 illustrates an example of a hybrid NDT system (acoustic emission, acousto-ultrasonic guided waves, digital image correlation, infrared thermography) used for evaluating fatigue damage and the residual lifetime of carbon–epoxy composite material. The acoustic emission consisted of two piezoelectric sensors (175–200 kHz) mounted in a straight line and centred on the surface of the sample with a distance between their centres of 120 mm. Firstly, these sensors allow only detection of evolving damage under the fatigue loading, whereas, at the end of each applied fatigue block (Figure 10), the same sensors were used to detect the material response signals (acousto-ultrasonic guided waves) due to pencil breaks operated in the centre of each 5 mm^2^ of a defined 120 × 20 mm^2^ area of interest [86]. The thermal field dissipated by the sample under fatigue loading was measured by an infrared camera (320 × 240 pixels with 10 mK sensitivity) oriented perpendicular to the surface of the sample. Finally, the fatigue strain full field was measured by the digital image correlation method using two CCD (2048 × 2048 pixels) cameras in stereoscopic mode.

**Figure 11:**
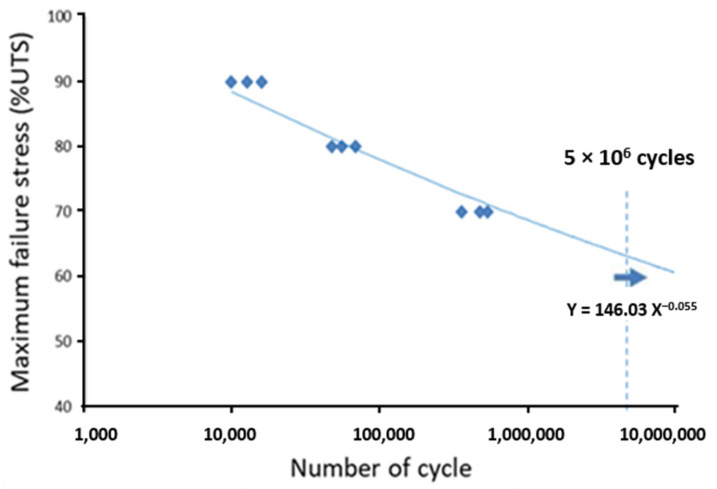
Experimental Wöhler curve (also known as S–N curve). Adapted from [21].

**Figure 12:**
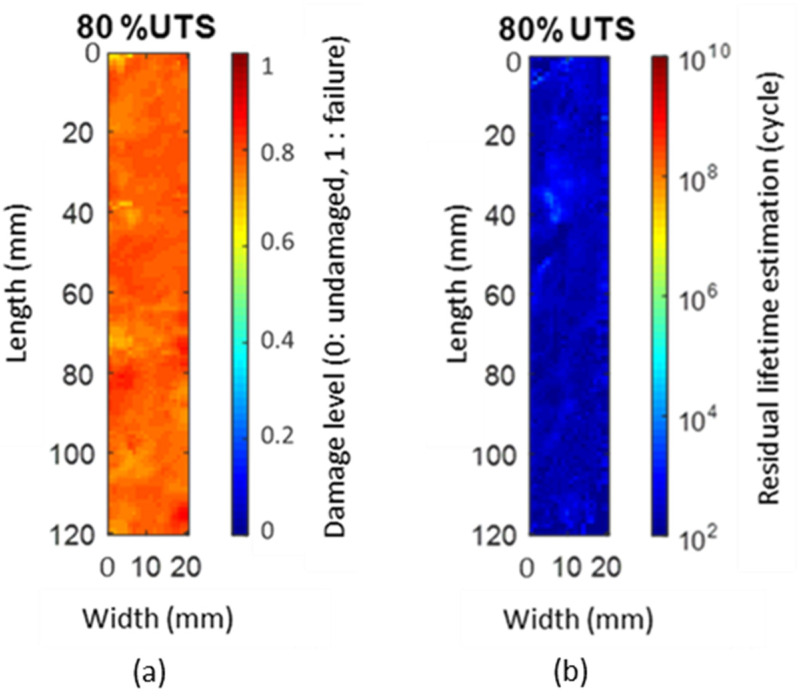
(**a**) Damage level and (**b**) residual lifetime estimated by neural network algorithm. Adapted from [21].

The data obtained from these NDT techniques were post-processed and homogeneously conditioned as 2D images in order to merge them (data fusion) by an artificial neural network algorithm [21]. Moreover, the implementation of this monitoring made it possible to collect enough data, e.g., the sufficient quantity which is necessary for the learning, validation test and execution of the material lifetime prognosis. The predicted lifetimes correlate with those obtained experimentally by the Wöhler curve (see Figure 11 with the error being less than 1% for all applied fatigue stress levels [21]). Figure 12 illustrates an example of the results obtained by the neural network algorithm for evaluation of the material damage level and prognosis of its residual lifetime after being submitted to a fatigue stress loading at 80% of the ultimate tensile strength (UTS). At this loading level, an overall damage rate of 0.8 is obtained, demonstrating the relevance of the results. Furthermore, the residual lifetime at this loading is low, estimated at around 10,000 cycles by the algorithm.

## 6. Trends and Perspectives

Conventional NDT techniques generally involve complex and time-consuming procedures, namely when setting up measurement devices. Data processing and results interpretation are also very labour intensive, sometimes leading to operator-subjective decisions. Moreover, most of these conventional techniques are independent of the objects to be inspected (external way) and are implemented only occasionally or periodically using sensors and other data acquisition equipment that are expensive, large and wire connected.

Current trends in the composite NDT community focus on a Structural Health Monitoring (SHM) approach, which consists of using embedded sensors–actuators for in situ and permanent inspection to overcome unpredictable failures through advanced warnings, with the purpose of reducing maintenance costs [87,88,89,90,91,92,93,94]. The most commonly embedded sensors used for this purpose are fibre-optic sensors, such as Fibre Bragg Gratings (FBGs) or chirped FBGs (CFBGs) [87], or piezoelectric sensors [88,89,90,91,92,93], which can even be integrated into a wireless sensor network autonomously powered by an energy harvester [89]. Implementation of such approaches needs firstly to define the type, number and location of sensors–actuators with a healthy process of integration into the material. Secondly, it should design appropriate acquisition and data storage systems depending on wired or wireless sensors where low power consumption solutions are required. Finally, a complete SHM system also comprises algorithms for signal processing and data-driven modelling (machine learning, deep learning, etc.) for, respectively, damage mechanism localisation and classification, and knowledge extraction, to predict the coming behaviour and residual lifetime of composite structures using supervised or unsupervised data mining algorithms [94].

**Figure 13:**
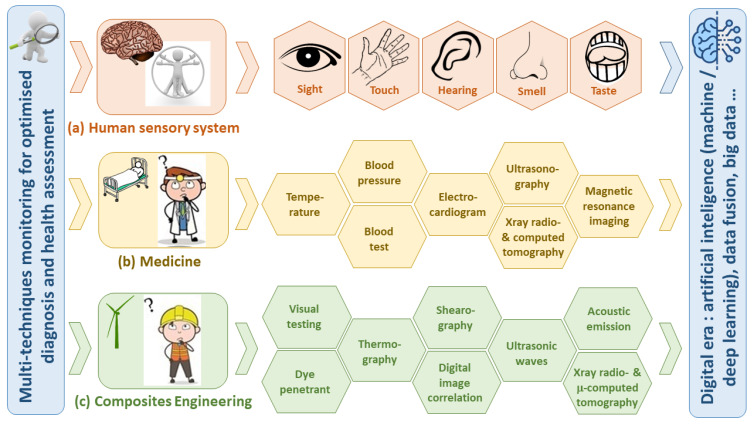
Comparison of “health” assessment in (**a**) nature, (**b**) medicine, and (**c**) materials engineering.

For several years now, as illustrated in Figure 13, material engineering and the NDT community have been inspired by medical practices, such as the use of X-ray radiography or tomography (medical scanner), ultrasound echography, temperature measurement, etc. (see Figure 13b). This inspiration will definitely continue in the coming years; firstly, in terms of practices, since medicine has already leaped into artificial intelligence several years ago (in the 1980s) initially for image analysis, robot-assisted surgery, etc. Indeed, the five senses (taste, sight, touch, smell and hearing) are functions by which humans identify external objects through the five inherent sensory organs: tongue, eye, hand, nose and ear, and their connection with the brain (see Figure 13a). Thanks to previous learning, the human brain manages to recognise perfectly the external objects when perceived by several sensory organs. Perception by a single organ is sometimes insufficient and can lead to misidentification of the object. Knowingly, medicine, which is intrinsically concerned with these sensory processes, has inspired them to improve clinical examinations by relying on complementary examinations (biochemical analysis of blood, radiology, ultrasounds, etc.).

Current and future technological and scientific advances in the digital field (digitalisation and computing capacity) and data science (big data, machine learning, deep learning, etc.) open up many possibilities for development in the SHM domain. Besides 3D imaging and numerical simulation, which are still likely to progress by taking advantage of digital advances, Artificial Intelligence (AI) is a very promising tool for SHM and predictive maintenance, which will make streamlining the diagnosis and decision-making possible.

**Figure 14:**
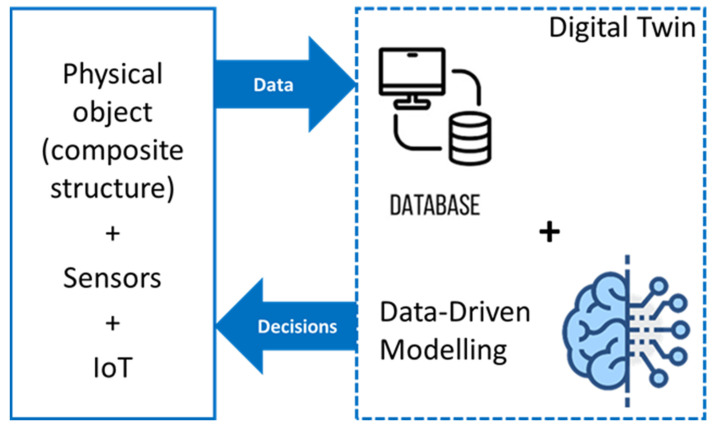
Main components for building digital twins for diagnosis of a physical object (ex. composite structure).

In the digital era, SHM implementation will be inspired even more by the sensory organs (~sensors), the nervous system (~sensor network connections) and the human brain (AI) functioning for more diagnosis efficiency and more autonomy and rationality in decision-making (see Figure 13c). Hence, the trend will be the use of more, different and connected wireless sensors to obtain a complete dataset, which will be managed by an IoT system and analysed by an AI algorithm for real-time diagnosis and predictive maintenance. This concept is newly known as Digital Twin, which is an emerging technology consisting of conducting interactive relationships between a physical object and its digital clone (Figure 14) [94,95,96]. With digital twin technology, diagnosis of composite materials and structures could be efficiently performed, their remaining lifetimes could be concurrently estimated, and extreme or complex scenarios of loading could be simulated and its impact predicted allowing for the enrichment of the dataset.

Additionally, SHM could benefit from Virtual Reality (VR) technology, as time-consuming field trips and dangerous site inspections of large infrastructures by teams of experts could be replaced by “virtual visits” operated through a VR platform [97].

## 7. Conclusions

The main non-destructive testing (NDT) techniques for surface and deep inspection of composite materials were reviewed and their advances and limitations were briefly highlighted. As a matter of fact, no single NDT method is completely sufficient nor false-negative or false-positive free. This is the reason why more and more studies are implementing a hybrid NDT approach to gather complementary information in order to improve the diagnosis of the damage state of materials. Such a hybrid (multi-techniques) procedure and its benefits for the complete non-destructive characterisation of composite materials were illustrated with an example.

In the digital era, current research efforts of the NDT-composite community focus on the Structural Health Monitoring (SHM) concept using embedded sensors–actuators and artificial intelligence algorithms for in situ, permanent and real-time inspection of structures. This community has drawn much inspiration from medical practices in the past, and in the coming years, will definitely continue taking advantage of recent advances in data mining (artificial intelligence, machine learning, deep learning, etc), Internet of Things (IoT) technologies, wireless and miniature sensors–actuators and the digital twin concept.

## Figures and Tables

**Table 1:** Comparison of different surface non-destructive techniques.

NDT Techniques	Sought Flaws						Advantages	Limitations
	Porosity	Fibre Misalignment	Matrix Cracks	Fibre Breakage	Delamination	Impact Damage		
Visual inspection	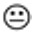	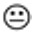	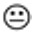	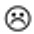	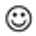	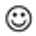	Inexpensive Simple and rapid	Depth indication missing
Dye penetrant (liquid penetrant)	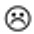	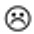	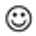	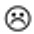	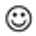	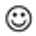	Inexpensive Simple and rapid	Depth indication missing Chemical wastewater
Infrared thermography	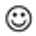	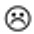	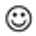	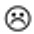	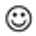	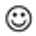	Eco-friendly Contactless inspection	Expensive Depth indication missing
Shearography	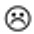	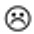	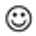	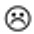	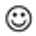	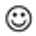	Contactless inspection High sensitivity	Expensive Roughness surface sensitive
Digital image correlation	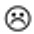	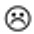	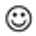	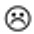	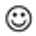	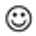	Contactless Inspection Full-field measurement	Speckle pattern needed Light conditions sensitive

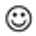
: Application proved. 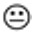: Application limited. 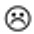: Not applicable.

**Table 2:** Comparison of different volume non-destructive techniques.

NDT Techniques	Sought Flaws						Advantages	Limitations
	Porosity	Fibre Misalignment	Matrix Cracks	Fibre Breakage	Delamination	Impact Damage		
Ultrasonic waves	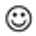	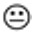	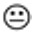	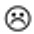	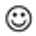	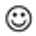	Detection, localisation, sizing One side is sufficient	Strong attenuation Complex interpretation
Acoustic Emission	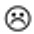	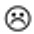	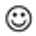	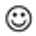	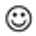	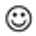	Real-time Detection, localisation Structural health monitoring	Only growing flawsNeed of loading Global method
X-ray radiography	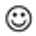	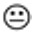	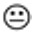	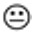	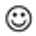	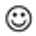	Reliability Imaging method	Expensive Ionising radiation
X-ray tomography	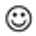	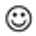	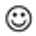	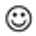	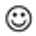	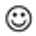	Reliability Imaging method 3D analysis	Expensive Ionising radiation Semi-destructive Laboratory method

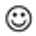
: Application proved. 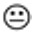: Application limited. 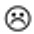: Not applicable.

## Data Availability

Not applicable.

## References

[1] Cuadra J., Vanniamparambil P.A., Hazeli K., Bartoli I., Kontsos A. (2013). Damage quantification in polymer composites using a hybrid NDT approach. Compos. Sci. Technol..

[2] Chaki S., Harizi W., Bourse G., Ourak M. (2015). Multi-technique approach for non-destructive diagnostic of structural composite materials using bulk ultrasonic waves, guided waves, acoustic emission and infrared thermography. Compos. Part A Appl. Sci. Manuf..

[3] Duchene P., Chaki S., Ayadi A., Krawczak P. (2018). A review of non-destructive techniques used for mechanical damage assessment in polymer composites. J. Mater. Sci..

[4] Christina D.R., Bruce L.B., Moore P.O. (1993). Aerospace applications of visual testing. Nondestructive Testing Handbook, Visual Testing Volume 9.

[5] Bossi R., Giurgiutiu V. (2015). Nondestructive testing of damage in aerospace composites. Polymer Composites in the Aerospace Industry.

[6] Zhong S., Nsengiyumva W. (2022). Visual Testing for Fiber-Reinforced Composite Materials. Nondestructive Testing and Evaluation of Fiber-Reinforced Composite Structures.

[7] Fotouhi S., Pashmforoush F., Bodaghi M., Fotouhi M. (2021). Autonomous damage recognition in visual inspection of laminated composite structures using deep learning. Compos. Struct..

[8] Graves W., Aminfar K., Lattanzi D. (2022). Full-Scale Highway Bridge Deformation Tracking via Photogrammetry and Remote Sensing. Remote Sens..

[9] Kergomard Y.D., Renard J., Thionnet A., Landry C. (2010). Intralaminar and interlaminar damage in quasi-unidirectional stratified composite structures: Experimental analysis. Compos. Sci. Technol..

[10] Kamar N.T., Hossain M.M., Khomenko A., Haq M., Drzal L.T., Loos A. (2015). Interlaminar reinforcement of glass fiber/epoxy composites with graphene nanoplatelets. Compos. Part A Appl. Sci. Manuf..

[11] Ismail M.F., Sultan M.T., Hamdan A., Shah A.U., Jawaid M. (2019). Low velocity impact behaviour and post-impact characteristics of kenaf/glass hybrid composites with various weight ratios. J. Mater. Res. Technol..

[12] Shipway N.J., Barden T.J., Huthwaite P., Lowe M.J.S. (2019). Automated defect detection for Fluorescent Penetrant Inspection using Random Forest. NDT E Int..

[13] Shipway N.J., Huthwaite P., Lowe M.J.S., Barden T.J. (2019). Performance Based Modifications of Random Forest to Perform Automated Defect Detection for Fluorescent Penetrant Inspection. J. Nondestruct. Eval..

[14] Harizi W., Chaki S., Bourse G., Ourak M. (2014). Mechanical damage assessment of Glass Fiber-Reinforced Polymer composites using passive infrared thermography. Compos. Part B Eng..

[15] Zalameda J., Winfree W. (2018). Detection and Characterization of Damage in Quasi-Static Loaded Composite Structures Using Passive Thermography. Sensors.

[16] Kelkel B., Popow V., Gurka M. (2020). Inline quantification and localization of transverse matrix cracking in cross-ply CFRP during quasi-static tensile testing by a joint event-based evaluation of acoustic emission and passive IR thermography. Compos. Sci. Technol..

[17] Popow V., Vogtmann J., Gurka M. (2022). In-situ characterization of impact damage in carbon fibre reinforced polymers using infrared thermography. Infrared Phys. Technol..

[18] La Rosa G., Risitano A. (2000). Thermographic methodology for rapid determination of the fatigue limit of materials and mechanical components. Int. J. Fatigue.

[19] Montesano J., Fawaz Z., Bougherara H. (2013). Use of infrared thermography to investigate the fatigue behavior of a carbon fiber reinforced polymer composite. Compos. Struct..

[20] Montesano J., Fawaz Z., Bougherara H. (2015). Non-destructive assessment of the fatigue strength and damage progression of satin woven fiber reinforced polymer matrix composites. Compos. Part B Eng..

[21] Duchene P. (2018). Nondestructive Characterization of Composite Materials under Fatigue Loading: Structural Health Diagnosis and Remaining Useful Life Prognostic Using Artificial Neural Networks. Ph.D. Thesis.

[22] Ardebili A., Alaei M.H. (2022). Non-destructive testing of delamination defects in GFRP patches using step heating thermography. NDT E Int..

[23] Harizi W., Chaki S., Bourse G., Ourak M. (2014). Mechanical damage assessment of Polymer–Matrix Composites using active infrared thermography. Compos. Part B Eng..

[24] Chen D., Wu N., Zhang Z. (2012). Defect Recognition in Thermosonic Imaging. Chin. J. Aeronaut..

[25] Li Y., Yang Z.-W., Zhu J.-T., Ming A.-B., Zhang W., Zhang J.-Y. (2016). Investigation on the damage evolution in the impacted composite material based on active infrared thermography. NDT E Int..

[26] Meola C., Carlomagno G.M., Squillace A., Vitiello A. (2006). Non-destructive evaluation of aerospace materials with lock-in thermography. Eng. Fail. Anal..

[27] Jinlong G., Junyan L., Fei W., Yang W. (2015). Inverse heat transfer approach for nondestructive estimation the size and depth of subsurface defects of CFRP composite using lock-in thermography. Infrared Phys. Technol..

[28] Saeed N., King N., Said Z., Omar M.A. (2019). Automatic defects detection in CFRP thermograms, using convolutional neural networks and transfer learning. Infrared Phys. Technol..

[29] Luo Q., Gao B., Woo W.L., Yang Y. (2019). Temporal and spatial deep learning network for infrared thermal defect detection. NDT E Int..

[30] Hung Y.Y., Ho H.P. (2005). Shearography: An optical measurement technique and applications. Mater. Sci. Eng. R Rep..

[31] Francis D., Tatam R., Groves R.M. (2010). Shearography technology and applications: A review. Meas. Sci. Technol..

[32] Zhang L., Tham Z.W., Chen Y.F., Tan C.Y., Cui F., Mutiargo B., Ke L. (2022). Defect imaging in carbon fiber composites by acoustic shearography. Compos. Sci. Technol..

[33] Hung Y.Y., Chen Y.S., Ng S.P., Liu L., Huang Y.H., Luk B.L., Ip R.W.L., Wu C.M.L., Chung P.S. (2009). Review and comparison of shearography and active thermography for nondestructive evaluation. Mater. Sci. Eng. R Rep..

[34] Taillade F., Quiertant M., Benzarti K., Aubagnac C. (2011). Shearography and pulsed stimulated infrared thermography applied to a nondestructive evaluation of FRP strengthening systems bonded on concrete structures. Constr. Build. Mater..

[35] De Angelis G., Meo M., Almond D.P., Pickering S.G., Angioni S.L. (2012). A new technique to detect defect size and depth in composite structures using digital shearography and unconstrained optimization. NDT E Int..

[36] Tao N., Anisimov A.G., Groves R.M. (2021). Shearography non-destructive testing of thick GFRP laminates: Numerical and experimental study on defect detection with thermal loading. Compos. Struct..

[37] Tao N., Anisimov A.G., Groves R.M. (2022). FEM-assisted shearography with spatially modulated heating for non-destructive testing of thick composites with deep defects. Compos. Struct..

[38] Li W., Wang D., Wu S. (2022). Simulation Dataset Preparation and Hybrid Training for Deep Learning in Defect Detection Using Digital Shearography. Appl. Sci..

[39] Aparna M.L., Chaitanya G., Srinivas K., Rao J.A. (2015). Fatigue Testing of Continuous GFRP Composites Using Digital Image Correlation (DIC) Technique a Review. Mater. Today Proc..

[40] Mahal M., Blanksvärd T., Täljsten B., Sas G. (2015). Using digital image correlation to evaluate fatigue behavior of strengthened reinforced concrete beams. Eng. Struct..

[41] Janeliukstis R., Chen X. (2021). Review of digital image correlation application to large-scale composite structure testing. Compos. Struct..

[42] Comer A.J., Katnam K.B., Stanley W.F., Young T.M. (2013). Characterising the behaviour of composite single lap bonded joints using digital image correlation. Int. J. Adhes. Adhes..

[43] Lima R.A.A., Perrone R., Carboni M., Bernasconi A. (2021). Experimental analysis of mode I crack propagation in adhesively bonded joints by optical backscatter reflectometry and comparison with digital image correlation. Theor. Appl. Fract. Mech..

[44] Giancane S., Panella F.W., Nobile R., Dattoma V. (2010). Fatigue damage evolution of fiber reinforced composites with digital image correlation analysis. Procedia Eng..

[45] Montesano J., Selezneva M., Levesque M., Fawaz Z. (2015). Modeling fatigue damage evolution in polymer matrix composite structures and validation using in-situ digital image correlation. Compos. Struct..

[46] Guo R., Xian G., Li C., Huang X., Xin M. (2021). Effect of fiber hybridization types on the mechanical properties of carbon/glass fiber reinforced polymer composite rod. Mech. Adv. Mater. Struct..

[47] Wang Y., Luo Q., Xie H., Li Q., Sun G. (2022). Digital image correlation (DIC) based damage detection for CFRP laminates by using machine learning based image semantic segmentation. Int. J. Mech. Sci..

[48] Datta S.K., Shah A.H. (2019). Elastic Waves in Composite Media and Structures: With Applications to Ultrasonic Nondestructive Evaluation.

[49] Harizi W., Chaki S., Bourse G., Ourak M. (2015). Mechanical damage characterization of glass fiber-reinforced polymer laminates by ultrasonic maps. Compos. Part B Eng..

[50] Borum K.K. Evaluation of the quality of thick fibre composites using immersion and air-coupled ultrasonic techniques. Proceedings of the 9th European Conference on Non-destructive Testing (ECNDT 2006).

[51] Hsu D.K., Barnard D.J. (2006). Inspecting Composites with Airborne Ultrasound: Through Thick and Thin. AIP Conf. Proc..

[52] Li C., Pain D., Wilcox P.D., Drinkwater B.W. (2013). Imaging composite material using ultrasonic arrays. NDT E Int..

[53] Revel G.M., Pandarese G., Cavuto A. (2013). Advanced ultrasonic non-destructive testing for damage detection on thick and curved composite elements for constructions. J. Sandw. Struct. Mater..

[54] Sun J., Chong A.Y.B., Tavakoli S., Feng G., Kanfoud J., Selcuk C., Gan T.-H. (2019). Automated Quality Characterization for Composites Using Hybrid Ultrasonic Imaging Techniques. Res. Nondestruct. Eval..

[55] Su Z., Ye L., Lu Y. (2006). Guided Lamb waves for identification of damage in composite structures: A review. J. Sound Vib..

[56] Toyama N., Yashiro S., Takatsubo J., Okabe T. (2005). Stiffness evaluation and damage identification in composite beam under tension using Lamb waves. Acta Mater..

[57] Böhm R., Hufenbach W. (2010). Experimentally based strategy for damage analysis of textile-reinforced composites under static loading. Compos. Sci. Technol..

[58] Duernberger E., MacLeod C., Lines D., Loukas C., Vasilev M. (2022). Adaptive optimisation of multi-aperture ultrasonic phased array imaging for increased inspection speeds of wind turbine blade composite panels. NDT E Int..

[59] Zhang H., Peng L., Zhang H., Zhang T., Zhu Q. (2022). Phased array ultrasonic inspection and automated identification of wrinkles in laminated composites. Compos. Struct..

[60] Li C., He W., Nie X., Wei X., Guo H., Wu X., Xu H., Zhang T., Liu X. (2021). Intelligent damage recognition of composite materials based on deep learning and ultrasonic testing. AIP Adv..

[61] Meng M., Chua Y.J., Wouterson E., Ong C.P.K. (2017). Ultrasonic signal classification and imaging system for composite materials via deep convolutional neural networks. Neurocomputing.

[62] Chou H.Y., Mouritz A.P., Bannister M.K., Bunsell A.R. (2015). Acoustic emission analysis of composite pressure vessels under constant and cyclic pressure. Compos. Part A Appl. Sci. Manuf..

[63] Crivelli D., Guagliano M., Eaton M., Pearson M., Al-Jumaili S., Holford K., Pullin R. (2015). Localisation and identification of fatigue matrix cracking and delamination in a carbon fibre panel by acoustic emission. Compos. Part B Eng..

[64] Doan D.D., Ramasso E., Placet V., Zhang S., Boubakar L., Zerhouni N. (2015). An unsupervised pattern recognition approach for AE data originating from fatigue tests on polymer–composite materials. Mech. Syst. Signal Process..

[65] Masmoudi S., El Mahi A., Turki S. (2016). Fatigue behaviour and structural health monitoring by acoustic emission of E-glass/epoxy laminates with piezoelectric implant. Appl. Acoust..

[66] He Y., Li M., Meng Z., Chen S., Huang S., Hu Y., Zou X. (2021). An overview of acoustic emission inspection and monitoring technology in the key components of renewable energy systems. Mech. Syst. Signal Process..

[67] Godin N., Huguet S., Gaertner R. (2005). Integration of the Kohonen’s self-organising map and k-means algorithm for the segmentation of the AE data collected during tensile tests on cross-ply composites. NDT E Int..

[68] Kumar C.S., Arumugam V., Sengottuvelusamy R., Srinivasan S., Dhakal H. (2017). Failure strength prediction of glass/epoxy composite laminates from acoustic emission parameters using artificial neural network. Appl. Acoust..

[69] Harizi W., Chaki S., Bourse G., Ourak M. (2022). Damage mechanisms assessment of Glass Fiber-Reinforced Polymer (GFRP) composites using multivariable analysis methods applied to acoustic emission data. Compos. Struct..

[70] Tarnopol’Skii Y.M., Kulakov V.L. (2001). Tests Methods for Composites. Survey of Investigations Carried out in the PMI of Latvian Academy of Sciences in 1964–2000. Mech. Compos. Mater..

[71] Jasinien E., Raiutis R., Literis R., Voleiis A., Vladiauskas A., Mitchard D., Amos M. (2009). NDT of wind turbine blades using adapted ultrasonic and radiographic techniques. Insight Non-Destruct. Test. Cond. Monit..

[72] Silva W., Lopes R., Zscherpel U., Meinel D., Ewert U. (2021). X-ray imaging techniques for inspection of composite pipelines. Micron.

[73] Ibrahim M.E. (2014). Nondestructive evaluation of thick-section composites and sandwich structures: A review. Compos. Part A Appl. Sci. Manuf..

[74] Scott A.E., Mavrogordato M., Wright P., Sinclair I., Spearing S. (2011). In situ fibre fracture measurement in carbon–epoxy laminates using high resolution computed tomography. Compos. Sci. Technol..

[75] Rolland H., Saintier N., Robert G. (2016). Damage mechanisms in short glass fibre reinforced thermoplastic during in situ microtomography tensile tests. Compos. Part B Eng..

[76] Garcea S.C., Wang Y., Withers P. (2018). X-ray computed tomography of polymer composites. Compos. Sci. Technol..

[77] Mehdikhani M., Straumit I., Gorbatikh L., Lomov S. (2019). Detailed characterization of voids in multidirectional carbon fiber/epoxy composite laminates using X-ray micro-computed tomography. Compos. Part A Appl. Sci. Manuf..

[78] Wei Q., Leblon B., La Rocque A. (2011). On the use of X-ray computed tomography for determining wood properties: A review. Can. J. For. Res..

[79] Sket F., Enfedaque A., Alton C., González C., Molina-Aldareguia J.M., Llorca J. (2014). Automatic quantification of matrix cracking and fiber rotation by X-ray computed tomography in shear-deformed carbon fiber-reinforced laminates. Compos. Sci. Technol..

[80] Gong Y., Shao H., Luo J., Li Z. (2020). A deep transfer learning model for inclusion defect detection of aeronautics composite materials. Compos. Struct..

[81] Gong Y., Luo J., Shao H., Li Z. (2022). A transfer learning object detection model for defects detection in X-ray images of spacecraft composite structures. Compos. Struct..

[82] Helwing R., Hülsbusch D., Walther F. (2022). Deep learning method for analysis and segmentation of fatigue damage in X-ray computed tomography data for fiber-reinforced polymers. Compos. Sci. Technol..

[83] Goidescu C., Welemane H., Garnier C., Fazzini M., Brault R., Péronnet E., Mistou S. (2013). Damage investigation in CFRP composites using full-field measurement techniques: Combination of digital image stereo-correlation, infrared thermography and X-ray tomography. Compos. Part B Eng..

[84] Crupi V., Guglielmino E., Risitano G., Tavilla F. (2015). Experimental analyses of SFRP material under static and fatigue loading by means of thermographic and DIC techniques. Compos. Part B Eng..

[85] Munoz V., Valès B., Perrin M., Pastor M.L., Welemane H., Cantarel A., Karama M. (2016). Damage detection in CFRP by coupling acoustic emission and infrared thermography. Compos. Part B Eng..

[86] Duchene P., Chaki S., Krawczak P. (2018). Acousto-ultrasonic damage evaluation of carbon fibre composites using pencil lead break sources. Proceedings of the 18th European Conference on Composites Materials (ECCM18).

[87] Rito R.L., Crocombe A.D., Ogin S.L. (2017). Health monitoring of composite patch repairs using CFBG sensors: Experimental study and numerical modelling. Compos. Part A Appl. Sci. Manuf..

[88] Lampani L., Sarasini F., Tirillò J., Gaudenzi P. (2018). Analysis of damage in composite laminates with embedded piezoelectric patches subjected to bending action. Compos. Struct..

[89] Lampani L., Gaudenzi P. (2018). Innovative composite material component with embedded self-powered wireless sensor device for structural monitoring. Compos. Struct..

[90] Tuloup G., Harizi W., Aboura Z., Meyer Y., Khellil K., Lachat R. (2019). On the use of in-situ piezoelectric sensors for the manufacturing and structural health monitoring of polymer-matrix composites: A literature review. Compos. Struct..

[91] Andreades C., Meo M., Ciampa F. (2021). Fatigue testing and damage evaluation using smart CFRP composites with embedded PZT transducers. Mater. Today Proc..

[92] Carrino S., Maffezzoli A., Scarselli G. (2021). Active SHM for composite pipes using piezoelectric sensors. Mater. Today Proc..

[93] Rocha H., Semprimoschnig G., Nunes J.P. (2021). Sensors for process and structural health monitoring of aerospace composites: A review. Eng. Struct..

[94] Kong K., Dyer K., Payne C., Hamerton I., Weaver P.M. (2022). Progress and Trends in Damage Detection Methods, Maintenance, and Data-driven Monitoring of Wind Turbine Blades—A Review. Renew. Energy Focus.

[95] Hu W., Zhang T., Deng X., Liu Z., Tan J. (2021). Digital twin: A state-of-the-art review of its enabling technologies, applications and challenges. J. Intell. Manuf. Spec. Equip..

[96] Kalidindi S.R., Buzzy M., Boyce B.L., Dingreville R. (2022). Digital Twins for Materials. Front. Mater..

[97] Luleci F., Li L., Chi J., Reiners D., Cruz-Neira C., Catbas F.N. (2022). Structural Health Monitoring of a Foot Bridge in Virtual Reality Environment. Procedia Struct. Integr..

